# Acute physiological effects on macromolecule digestion following oral ingestion of the enzyme blend Elevase® in individuals that had undergone an ileostomy, but were otherwise healthy—a randomized, double blinded, placebo-controlled exploratory study

**DOI:** 10.3389/fnut.2024.1357803

**Published:** 2024-07-16

**Authors:** Shahneela Mazhar, Annie Simon, Joan Colom, Ekaterina Khokhlova, Martin Buckley, Christopher Phipps, John Deaton, Kieran Rea

**Affiliations:** ^1^ADM Cork H&W Limited, University College Cork, Cork, Ireland; ^2^Mercy University Hospital, Cork, Ireland; ^3^ADM Deerland Probiotics and Enzymes, Kennesaw, GA, United States

**Keywords:** Elevase®, enzyme supplement, ileostomy, macromolecule, digestion, randomized crossover clinical trial

## Abstract

Digestive enzymes can selectively degrade proteins, carbohydrates and lipids; and their supplementation alongside food may accelerate the breakdown of complex food matrices, facilitate greater nutrient absorption, decrease food sensitivities and aid in the management of certain disease states. Several intrinsic and extrinsic factors govern food digestion and for every individual this phenomenon is unique. This study was conducted as a randomized, crossover, placebo-controlled design where each participant served as their own control. This post-hoc analysis investigated the impact of a dietary enzyme supplementation blend known as Elevase® on dietary macromolecule digestion in samples from otherwise healthy participants that had previously undergone a small bowel resection, resulting in an ileostomy (NCT04489810). This is the first time this study-paradigm has been used for the assessment of *in vivo* dietary breakdown following enzyme supplementation. Arguably, this technique offers superior data when compared to that generated in artificial gut digestion models, preclinical animal models, or indeed conventional clinical studies using stool analyses, as it allows real-time access to samples *in situ* in the small intestine where the majority of nutritional absorption takes place. It was demonstrated that after 4 h, Elevase® significantly increased monosaccharide levels (predominantly glucose and fructose) in the ileostomy samples taken from the same individuals on the same diet on a different day. In addition, the bile salt taurohyodeoxycholic acid was also increased, suggesting a physiological host response to the macromolecule digestion induced by the enzymatic blend. Overall, these findings suggest Elevase® could accelerate food digestion and potentially increase nutrient availability from the diet.

## 1 Introduction

Naturally occurring digestive enzymes help catabolize food and aid digestion to release nutrients that govern metabolism and gut homeostasis. Along the gastrointestinal tract there are a number of anatomical loci including the mouth, stomach, pancreas, liver, gallbladder and intestines where specific digestive enzymes are released and activated. Food digestion is influenced by the physical form of the food, granular structure, surface area available to enzymes as well as factors specific to the individuals consuming the food such as chewing, transit time and the pH of stomach acid, circadian rhythm of hunger/satiety among others (1). The small intestine is responsible for around 90% of digestion and absorption of nutrients from the diet along the length of the duodenum, the jejunum, and the ileum (2). In the duodenum, secretion of enzymes, bile salts, and bicarbonate allow for neutralization of pH from the stomach to allow digestion of complex carbohydrates, proteins, and lipids. The jejunum, which follows the duodenum, is specialized in the absorption of the digested particles. Then, the ileum allows absorption of the remaining nutrients, such as vitamin B12 and reabsorption of bile salts. Some of the main native enzymes secreted during this process include amylase for the breakdown of carbohydrates and sugars; lipases for the breakdown of fats; proteases and peptidases for the breakdown of proteins (3); lactase for the breakdown of lactose (4), and sucrase for the breakdown of sucrose (5). Digestive enzyme supplements have gained popularity for their benefits in alleviating gut irritation, heartburn, food sensitivity and other ailments as well as improving digestion (3, 6). Various formulations of enzyme supplements are available as food/dietary supplements and have been studied for bloating, diarrhea, gas, weight loss and food sensitivities among others (3, 7).

However, there is a limited understanding of the temporal profile of digestion of proteins, lipids and carbohydrates from different food matrices. In an artificial INFOGEST digestion model, it was recently demonstrated that Elevase® significantly enhanced protein hydrolysis and gluten breakdown at the gastric stage, and that this enhanced digestion was maintained into the simulated intestinal environment (8). Elevase® is composed of 13 enzymes namely alpha-galactosidase, amylase, beta-glucanase, cellulase, diastase, endo-peptidase complex, exo-peptidase complex, glucoamylase, invertase, lactase, lipase, protease and xylanase derived from *Aspergillus*, *Trichoderma*, *Saccharomyces*, *Candida*, and *Bacillus* microbes (Table 1).

**Table 1:** Composition of the supplemental enzyme mix.

Component	Source	Units	Amount (mg)
Alpha Galactosidase	Aspergillus niger	150 GalU^1^	-
Amylase	Aspergillus oryzae	1,200 DU^2^	-
Beta glucanase	Trichoderma longibrachiatum	15 BGU^3^	-
Cellulase	Trichoderma longibrachiatum	500 CU^4^	-
Diastase	Aspergillus oryzae	1,200 DP^5^	-
Endo-peptidase complex	Aspergillus niger, Bacillus sp.	75,000 HUT^6^/500 SAPU^7^	-
Exo-peptidase complex	Aspergillus oryzae	125 DPPIV^8^	-
Glucoamylase	Aspergillus niger	5 AGU^9^	-
Invertase	*Saccharomyces cerevisiae*	100 SU^10^	-
Lactase	Aspergillus oryzae	500 ALU^11^	-
Lipase	*Candida rugosa*	500 FIP^12^	-
Protease	Bacillus sp.	5,500 PC^13^	-
Rice dextrin	-	-	79.8
Rice Bran	-	-	25.0
Xylanase	Trichoderma longibrachiatum	500 XU^14^	-

Components of the enzyme-based preparation including the enzymes and their sources, the total enzymatic activity of each enzyme in one tablet and the definition of the enzyme units used as provided by the manufacturer. ^1^GalU: α-Galactosidase Units – One unit is the quantity of the enzyme that will liberate p-nitrophenol at the rate of 1 μmol/min under the conditions of the assay (pH 5.5 and 37°C). ^2^DU: α-amylase dextrinizing unit – One unit is the quantity of α-amylase that will dextrinize soluble starch in the presence of an excess of β-amylase at the rate of 1 g/h at 30°C. ^3^BGU: β-Glucanase Units – One β-glucanase unit is defined as that quantity of enzyme that will liberate reducing sugar (as glucose equivalence) at a rate of 1 μmol/min under the conditions of the assay (pH 6.5 and 40°C). ^4^CU: Cellulase Units – One cellulase unit is defined as the amount of activity that will produce a relative fluidity change of 1 in 5 min in a defined carboxymethyl cellulose substrate under the conditions of the assay (pH 4.5 and 40°C). ^5^DP°: Diastase Units – One unit of diastase activity, expressed as degrees diastatic power, is defined as that amount of enzyme contained in 0.1 mL of a 5% solution of the sample enzyme preparation that will produce sufficient reducing sugars to reduce 5 mL of Fehling’s solution when the sample is incubated with 100 mL of the substrate for 1 h at 20°C. ^6^HUT: Hemoglobin Unit Tyrosine base – One HUT unit of proteolytic activity is defined as that amount of enzyme that produces a hydrolysate whose absorbance at 275 nm is the same as that of a solution containing 1.10 μg/mL of tyrosine in 0.006 N hydrochloric acid in 1 min under the conditions of the assay (pH 4.7 and 40°C). ^7^SAPU: Spectrophotometric acid protease units – One spectrophotometric acid protease unit is that activity that will liberate 1 μmol of tyrosine per minute under the conditions specified (pH 3.0 and 37°C). ^8^DPPIV: Dipeptidyl peptidase units: One unit will produce 1.0 μmole of p-nitroaniline from Gly-L-Pro p-nitroanilide per minute in 100 mM Tris–HCl under the conditions of the assay (pH 7.6 at 37°C). ^9^AGU: Glucoamylase Units – One unit of glucoamylase activity (Amyloglucosidase) is defined as the amount of glucoamylase that will liberate 0.1 μmol/min of p-nitrophenol from the p-nitrophenyl-α-Dglucopyranoside (PNPG) solution under the conditions of the assay (pH 4.3 and 50°C). ^10^SU: Sumner Units – One unit is the quantity of enzyme which will convert 1 mg of sucrose to glucose and fructose in 5 min under the conditions of the assay (pH 4.5 and 20°C). ^11^ALU: Lactase Units – One unit is defined as that quantity of enzyme that will liberate o-nitrophenol at a rate of 1 μmol/min under the conditions of the assay (pH 4.5 and 37°C). ^12^FIP: One unit of enzyme activity is defined as that quantity of a standard lipase preparation (Fungi Lipase-International FIP Standard) that liberates the equivalent of 1 μmol of fatty acid per minute from the substrate emulsion under the described assay conditions (pH 7.00 and 37°C). ^13^PC: Bacterial Protease Units – One unit is defined as that quantity of enzyme that produces the equivalent of 1.5 μg/mL of Ltyrosine per minute under the conditions of the assay (pH 7.0 and 37°C). ^14^XU: Xylanase units – One unit is defined as the amount of enzyme which liberates 1 μmol of xylose per minute under the conditions of the assay (pH 5.3 and 50°C). Enzymatic activity unit definitions are based on the Food Chemicals Codex. Detailed information about each assay can be found in this publication (National Research Council 1996. Food Chemicals Codex: Fourth Edition).

This exploratory post-hoc analysis study was part of a larger placebo-controlled, crossover, exploratory clinical study looking at the impact of dietary supplements on the structural properties of food, gluten digestion and food digestion. Herein, we investigated whether the enzyme blend Elevase® enhances macronutrient metabolism in a simple diet. Participants included individuals with an ileostomy but who were otherwise healthy and who have been stable for at least 3 months post-surgery and exhibited normal stoma function (9). This approach takes into consideration the many intrinsic and extrinsic factors associated with digestion and allows real-time and direct access to the intestinal contents from the terminal ileum, following the administration of dietary enzymes. To our knowledge, this is the first clinical trial showing the effect of dietary enzymes on food digestion from ileum samples.

## 2 Materials and methods

### 2.1 Study design

**Figure 1:**
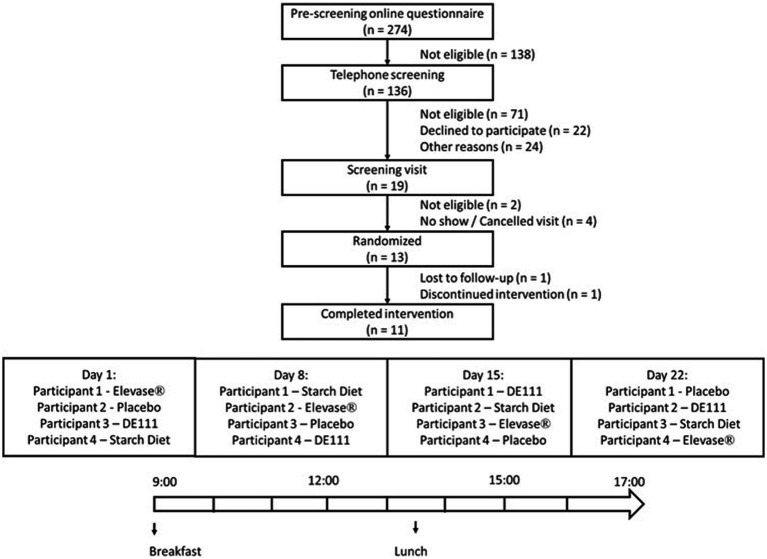
Flowchart of the study profile and example of treatment schedule followed by participants. The graph shows the four arms, placebo, Elevase®, DE111®, and starch diet included in the study. This article only discusses results of placebo and Elevase® treatments.

This study is a post-hoc analysis of a subset of data from a larger randomized, double-blind, placebo-controlled trial with four arms (placebo, Elevase®, probiotic DE111® and a modified starch diet) in individuals that had undergone ileostomy procedure, but were otherwise healthy (Figure 1). The overall trial (original study) was conducted according to the Declaration of Helsinki of 1975 and ICH-Good Clinical Practice (GCP) guidelines, revised in 2013; approved by the Clinical Research Ethics Committee of the Cork Teaching Hospitals (Review Reference Number: ECM 4 (d) 05/05/2020) and registered on clinicaltrials.gov (NCT04489810). The trial was conducted between October 2020 and April 2021 at a single site in Ireland.

A total of 13 participants were enrolled, with 11 participants (7 males and 4 females) completing this study (Figure 1). Each participant attended the clinic on 4 separate days spaced 1 week apart and received a different intervention on each day. The allocation sequence was generated using simple randomization. The sequence was concealed and implemented so that the researchers and participants remained blinded to treatment.

The age range of male participants was 24 to 75 years, with a BMI range of 22.92 to 29.94. Female participants had an age range of 40 to 58 years and a BMI range of 21.36 to 29.64 (Table 2). Participants were included in the trial based on the following criteria: having a stable ileostomy for the preceding the 3 months, demonstrating normal stoma function and being otherwise healthy. Exclusion criteria included obstruction of the stoma in the previous 3 months, body mass index below 18 kg/m^2^ or above 30 kg/m^2^, being immunocompromised, history of bariatric surgery, history of drug and/or alcohol abuse, concurrent participation in other research studies, planning on conceiving during the study or being pregnant. In addition, a current or past diagnosis of one or more of the following was also an exclusion criterion: coeliac disease, allergy to wheat and/or any other ingredients in the study meals; mouth, throat or active gastrointestinal pathology (other than ileostomy) that may affect normal ingestion and digestion of food; pancreatic disease; diabetes (Type 1 and Type 2). Participants were asked not to use any proton pump inhibitors or anti-diarrheal medication during the week and day, respectively, preceding each study day. They were also asked to refrain from excessive alcohol consumption (more than 12 units (unit is defined as 10 mL or 8 g of pure alcohol) per week) and intensive physical activity the day before the study sessions. All participants gave their written informed consent to participate after receiving verbal and written information about the research, patient information leaflet, and consented to the inclusion of their anonymized data in this publication.

**Table 2:** Baseline characteristics of participants recruited in this study, categorized by gender.

Gender	Age range	Temperature range (°C)	Systolic BP range (mmHg)	Diastolic BP range (mmHg)	Pulse Rate range (bpm)	Weight range (kg)	Height range (m)	BMI range
Male	24–75	36.4–37.1	114–160	69–87	56–90	71.4–97	1.76–1.8	22.92–29.94
Female	40–58	36.6–37.1	103–124	63–81	73–76	61–82.4	1.58–1.69	21.36–29.64

The table includes ranges of age, temperature, systolic and diastolic blood pressure, pulse rate, weight, height, and BMI for male and female participants.

Participants were advised to consume gluten-free dinner no later than 21 h00 the evening before each study session, and this was confirmed by oral consent on the test study day. Only water was allowed after the gluten-free dinner. The following morning, participants arrived at the study site in a fasted state and remained on site for the duration of the study session. Each participant underwent four separate one-day treatment regimens spaced one week apart, that were identical in design except for the intervention. Every participant received either placebo or Elevase® on 2 of the 4 visit days, with minimum seven days washout in between treatments designed to avoid carry-over effects. Participants emptied their ileostomy pouch into a sample collection bag prior to consumption of the investigational products and standard breakfast [two pots of porridge (Flahavan’s Organic Original Porridge, Kilmacthomas, Ireland)], one whole-grain Weetabix biscuit (Burton Latimer, UK) at 09 h00. This was the same breakfast by all participants on each study day. The chemical composition for the standard breakfast is presented in Table 3. A standard lunch, at 13 h30, consisting of 400 g of a smooth soup (Cully & Sully, Cork, Ireland) and 150 g of jelly (Boyne Valley Group, Louth, Ireland) was consumed by all participants. Throughout the study session, participants’ water intake was monitored but unrestricted (up to a maximum 1.5 L each session). Ileal effluent was collected at baseline and once every hour for 8 h after breakfast. Participants answered a basic questionnaire at the end of each session to report any potential abdominal or epigastric symptoms.

**Table 3:** Chemical composition of the standard breakfast.

Meal	Ingredients	Nutrients	Concentration (100 g)
Flahavan’s organic original porridge	100% Organic wholegrain rolled oats	Fat	5.1
		Protein	13
		Carbohydrates	71
		Sugar	5.4
		Salt	0.02
		Fiber	5.6
Weetabix original	Wholegrain wheat (95%)	Fat	2.0
	Malted barley extract	Carbohydrates	69
	Sugar	Fiber	10
	Salt	Protein	12
	Niacin	Salt	0.28
	Iron	Niacin	0.014
	Riboflavin (B2)	Iron	0.012
	Thiamin (B1)	Riboflavin (B2)	0.0012
	Folic acid	Thiamin (B1)	0.00094
		Folic Acid	170 μg

Previous data on the DE111® intervention arm from this clinical study focused on germination of spores, and targeted metabolomics and proteomics and are published elsewhere (10, 11). Data on the modified starch diet are unpublished. The post-hoc analyses on the placebo and Elevase® arms were specifically aimed to assess the efficacy of Elevase® in metabolizing carbohydrate, lipid, and proteins in the ileal effluent when co-administered with a meal.

A flowchart of the study is depicted in Figure 1.

### 2.2 Study product

The study products were provided in the form of capsules packaged in identical containers in single servings. The Elevase® supplement was composed of alpha-galactosidase, amylase, beta-glucanase, cellulase, diastase, endopeptidase complex, exopeptidase complex, glucoamylase, invertase, lactase, lipase, protease, xylanase, rice dextrin and rice bran (Table 1). The placebo consisted of an identical capsule containing maltodextrin.

### 2.3 Ileal effluent collection and processing

Ileal effluent sample collection was performed on-site by the participants, who emptied their ileostomy pouches into sterile bags (Buerkle™ SteriBag™ StandUp Polyethylene Sampling Bags, Bad Bellingen, Germany). After sample collection, the participants placed the samples in a polystyrene box with frozen (−80°C) cooling packs prior to on-site processing. Samples were collected every hour for 8 h. Upon collection each sample was weighed, diluted 50:50 (w/w) with PBS (7.2–7.4 pH) and thoroughly homogenized by vigorous shaking to ensure a homogenous mixture. While different volumes were present at each timepoint between individuals, and even within individuals on different respective collection days (although to a lesser extent), each hourly sample was treated the same and mixed thoroughly.

### 2.4 Metabolomics

Semi-polar metabolites were detected by MS-Omics (Vedbæk, Denmark) as follows. Samples were diluted with 80 μL of 10% methanol prior to analysis. The study was carried out using a Thermo Scientific Vanquish Liquid Chromatography coupled to Thermo Q Exactive hybrid quadrupole-orbitrap mass spectrometer (Thermo Fisher, Roskilde, Denmark). An electrospray ionization interface was used as ionization source. Analysis was performed in negative and positive ionization mode. The UPLC was performed using a slightly modified version of the protocol described by Catalin et al. (12). Peak areas were extracted using Compound Discoverer 3.1 (Thermo Fisher, Roskilde, Denmark). Identification of compounds were performed at four levels; Level 1: identification by retention times (compared against in-house authentic standards), accurate mass (with an accepted deviation of 3 ppm), and MS/MS spectra, Level 2a: identification by retention times (compared against in-house authentic standards), accurate mass (with an accepted deviation of 3 ppm). Level 2b: identification by accurate mass (with an accepted deviation of 3 ppm), and MS/MS spectra, Level 3: identification by accurate mass alone (with an accepted deviation of 3 ppm). Before performing any statistical analysis, all area values were converted to Log2.

### 2.5 Analysis of macronutrients

In this study we examined the efficacy of Elevase® by employing a total of 11 commercially available kits that will quantify the levels of carbohydrate, protein, and lipid metabolites. Specifically, these kits assessed the levels of amylose (Cohesion Biosciences Cat # CAK1178), maltose (Cohesion Biosciences Cat # CAK1268), galactose (Cohesion Biosciences Cat # CAK1259), glucose (RayBiotech Cat# MA-GLU), fructose (Cohesion Biosciences Cat # CAK1035), cholesterol (Cohesion Biosciences Cat# CAK1116), triglycerides (Cohesion Biosciences Cat# CAK1086), glycerol (Cohesion Biosciences Cat # CAK1231) proteins (Abbexa Cat# abx090640), branched-chain amino acids (Cell Biolabs Cat# MET-5056) and total free amino acids (Cell Biolabs Cat# MET-5055).

#### 2.5.1 Sample preparation

Ileostomy samples were weighed (approximately 100 mg) and transferred to bead tubes and were resuspended in 1 mL of assay buffer per 100 mg. The samples were homogenized at 3500 rpm for 30 s using Beadbug homogenizer and placed in ice for 1 min between cycles. The process was repeated three times. The homogenized samples were centrifuged at 9000 x G for 15 min. The supernatants were recovered and stored at −20°C or assayed directly. All assay and calculations were performed according to the manufacturer’s instructions for all assay kits.

### 2.6 Statistical analysis

A formal power calculation was not performed for this analysis as it is a post-hoc evaluation of data from a larger pilot trial. All statistical analyses were performed using GraphPad Prism v 9.3.1 (San Diego, CA). The Shapiro–Wilk test was used to determine normal distribution of the data. A paired 2-tailed *t*-test was performed to determine if there were any difference between the Elevase® and placebo groups for the ELISA tests. For the mass spectrometry data, the data was normalized versus placebo control. The data were log-transformed and Pareto-scaled. Comparisons between groups were made using one-way ANOVA with Sidak multiple comparison *post hoc* test. All statistical analyses were performed using GraphPad Prism 9 (GraphPad Software Inc., San Diego, CA, USA).

## 3 Results

### 3.1 Elevase® induces changes in micronutrient concentrations 4 h after ingestion

In this study we assessed the impact of Elevase® on macronutrient (fat, protein and carbohydrate) digestion in ileostomy samples from study participants four hours after food digestion. After the completion of the trial, it was decided to only analyze the 4 h samples for the present Elevase® study. Three factors contributed to this decision; (1) one has to consider the ‘dead volume’ of the intestine where the passage of ileal effluent at earlier timepoint may not have allowed for the treatment to have any effect merely because the product would not have passed along the intestine until later timepoints, (2) the 4 h timepoint allowed for the longest Elevase® activity in the small intestine before lunch was served to the study participants, preventing interferences caused by this meal and physiological events relating to distension of the stomach or hormones associated with food being present in the stomach; (3) four hours allowed for enough digested food from the breakfast meal to be collected in the participants stomach, as the small-bowel transit time was determined to be 297 ± 65 min for men and 95% range of 154–440 min for women (13). However, samples collected from 3 participants had low biomass at 4 h timepoint, therefore, samples from only 8 participants were evaluated. Baseline information of participant recruited in this study are presented in Table 2.

**Figure 2:**
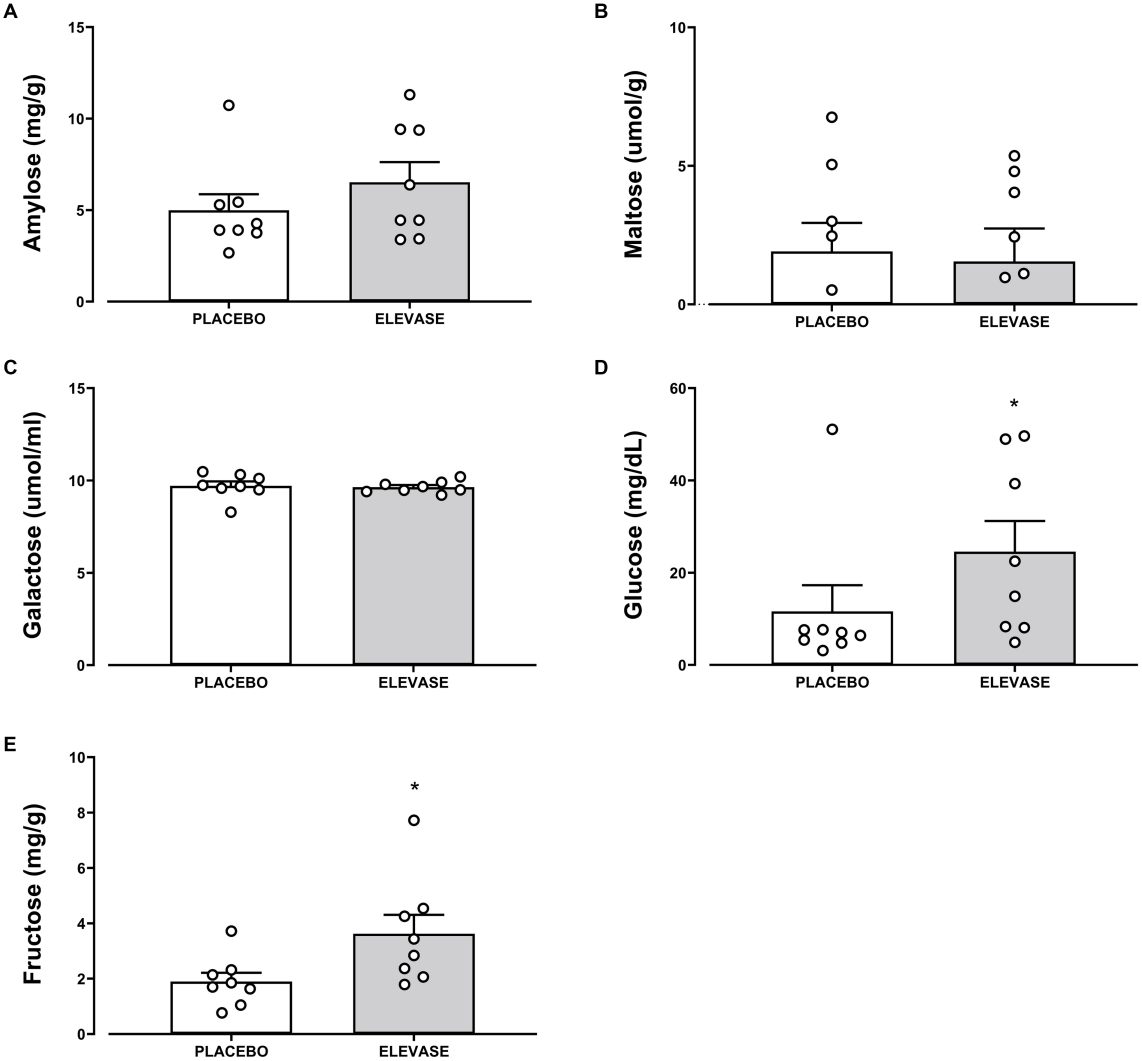
Elevase® had no significant effect on **(A)** amylose, **(B)** maltose, **(C)** galactose, with the exception of **(D)** glucose, and **(E)** fructose levels that were statistically significant in ileostomy samples from study participants 4 h after food ingestion.

**Figure 3:**
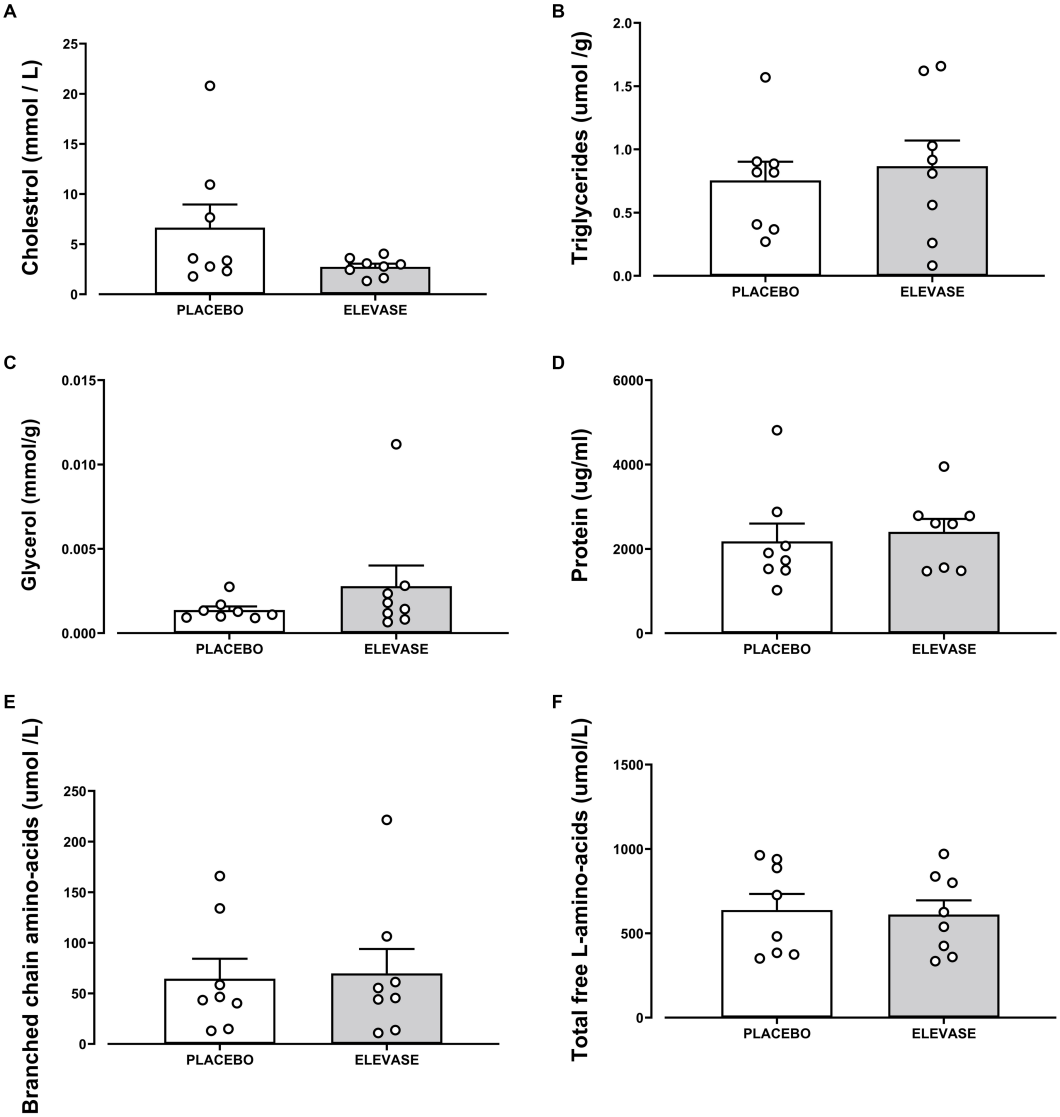
Elevase® had no significant effect on **(A)** cholesterol, **(B)** triglycerides, **(C)** glycogen, **(D)** protein, **(E)** branched-chain amino acid, and **(F)** free amino acid or levels in ileostomy samples from study participants 4 h after food ingestion.

The concentration of amylose (polysaccharide, main component of starch), maltose (disaccharide-a product of starch digestion), galactose, fructose, glucose (monosaccharides; final products of carbohydrate digestion), triglycerides (the main constituents of body fat and an index of blood glucose levels), glycerol (backbone of triglycerides), and cholesterol (a sterol important in cell membranes) were assessed as an index of carbohydrate and lipid digestion in the small intestine (Figures 2, 3). Elevase® significantly increased glucose [*t*(7) = 2.089, *p* = 0.0391] and fructose [*t*(7) = 2.126, *p* = 0.0234] levels in ileostomy samples from study participants 4 h after food ingestion, while having no significant effect on, amylose [*t*(7) = 0.9068, *p* = 0.250], maltose [*t*(7) = 0.2134, *p* = 0.3291], and galactose [*t*(7) = 0.3581, *p* = 0.730] at this timepoint (Figure 2). Elevase® had no significant effect on glycerol [*t*(7) = 1.072, *p* = 0.4609], triglycerides [*t*(7) = 0.3761, *p* = 0.718] or cholesterol (B) [*t*(7) = 1.741, *p* = 0.1253] concentration (Figure 3). Similarly, Elevase® had no significant effect on protein [*t*(7) = 0.3978, *p* = 0.7027], free amino acid [*t*(7) = 0.8913, *p* = 0.4023] or branched-chain amino acid levels [*t*(7) = 0.1911, *p* = 0.8239] in ileostomy samples from study participants 4 h after food ingestion (Figure 3).

### 3.2 Elevase® administration may be associated with changes in bile salt in the small intestine of humans 4 h after ingestion

**Figure 4:**
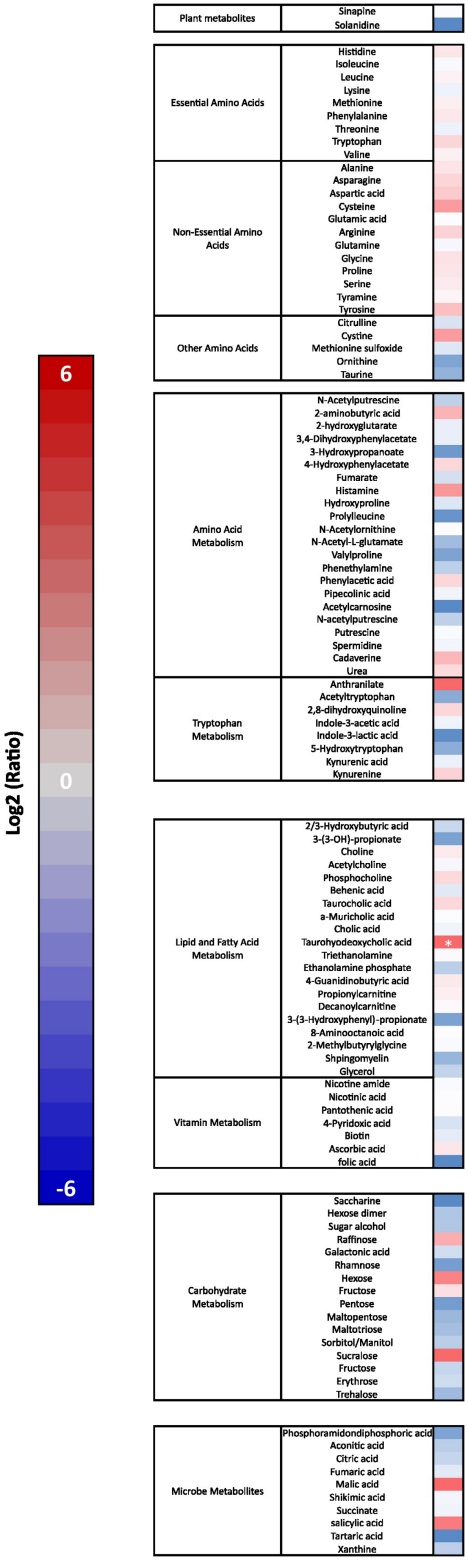
Elevase® effect on amino acid, lipid, carbohydrate, plant and microbial metabolites in the small intestine 4 h after ingestion. Results show Log2 average fold change (*n* = 8) in a heat map with a scale from red (increased levels) to blue (decreased levels) compared to placebo group at 4 h. Asterisk represents a significant effect of Elevase® as compared to placebo as determined by one-way ANOVA followed by Sidak multiple comparison *post hoc* test.

Mass spectrometry was used to determine metabolic changes induced by Elevase® compared to placebo in the small intestinal tract of the study participants [Figure 4, *F*(1, 658) = 13.55, *p* = 0.0003]. There was no significant difference in analytes associated with dietary plant metabolism, vitamin metabolism, amino-acid metabolism, carbohydrate metabolism and microbial metabolism remained unaltered. Similarly, there was no significant effect of Elevase® on essential and non-essential amino acids (Figure 4). Of all metabolites involved in lipid metabolism, only the bile salt taurohyodeoxycholic acid was significantly increased as compared to placebo after 4 h as determined by the one-way ANOVA followed by Sidak multiple comparison *post hoc* test (Figure 4).

## 4 Discussion

Food digestion is a multiphase, complex process which involves several organs and a variety of enzymes. Following the ingestion of food, the salivary enzymes, chewing, the acid pH of stomach and the activity of the gastric enzymes will initiate the digestion process and determine the degree of degradation of the food matrix before it enters the small intestinal tract (14, 15). This will vary between individuals as the level of chewing, enzymatic activity and temporal profile of gastric emptying are all person specific (16, 17). Consequently, the digestion of the food matrix along the duodenum, jejunum and ileum are further influenced by secretory enzymes, bile, bicarbonate and the temporal profile of movement of ingesta along the intestines, which again is unique to each individual. The process is also influenced by the physicochemical properties of the food being ingested and the surface area that is available for enzymatic degradation (18). External factors like stress can also have a significant impact on function and efficacy of the digestive process. Stress has been linked to alterations of stomach transit time and pH, intestinal absorption and permeability (19). Stress has been reported to affect as many as 25% (20) of the population worldwide. Therefore, it is possible that simultaneously sub-optimal digestion of food and nutrient absorption is occurring for part of the population. Digestive enzyme supplementation may represent an effective option to help impaired digestive process for better nutrient absorption, improving the prognosis for several digestive and malabsorption disorders and food sensitivities (3).

Studies to distinguish and quantitate the endogenous and dietary components of ileal chyme have relied on macronutrient-rich or-depleted diets, enzyme-hydrolyzed diet prior to ingestion, artificial digestion models, and isotope labeling of components of the diet or endogenous system among others (21).

In this study, we assessed the breakdown of carbohydrates (Figure 2), lipids (Figure 3) and proteins in ileal effluents (Figures 3, 4) of participants four hours after ingesting breakfast. The four-hour timepoint was selected as it allowed for gastric digestion and emptying into the ileum (13). Later time points were disregarded as lunch ingestion could have interfered with the enzymatic blend concentration and activity. Carbohydrates were the main dietary component of oats (71 g for each 100 g) (22) and Weetabix (69 g for each 100 g) served during the study breakfast. No significant difference was observed in the levels of galactose between the two treatment groups. One potential explanation for this is that this monosaccharide is not commonly produced following the digestion of wheat derived products. Molecules linked to the metabolism of complex carbohydrates such as amylose or maltose were not statistically significant between the groups. The reason for this could be the fact that amylose was broken down by the action of salivary and pancreatic amylases and Elevase® into glucose and maltose. Then, the presence of maltase in the small intestine would have further catabolized maltose to glucose. In other words, no difference was seen in the levels of these molecules between the two treatment groups because the combined activity of native digestive enzymes and the Elevase® led to the degradation of complex carbohydrates to glucose. However, in these samples, it was demonstrated that Elevase® significantly increased glucose and fructose concentration in ileostomy samples. This increase in glucose and fructose observed when participants took the Elevase® supplement suggests that the amylase, glucoamylase, β-glucanase, diastase, invertase, glucoamylase cellulase and xylanase present in the enzyme blend supported an accelerated digestion of the complex carbohydrates in oats and Weetabix to simple monosaccharides.

The presence of a bile salt was also increased in the individuals taking Elevase® (Figure 4). Bile salts are known to emulsify hydrophobic compounds and contribute to lipolysis facilitating absorption by enterocytes. Therefore, it is possible that the host response to an increase in lipid concentration due to Elevase® led to an increase in bile salts to further aid in their digestion (23). However, lipids tested in this study were not altered by the presence of the enzymatic mix, as triglycerides and cholesterol levels remained unchanged between both groups. Both oats and Weetabix have very low concentrations of fat [5.1 g and 2 g for each 100 g (22) respectively] relative to carbohydrates, which could explain the lack of change in lipid metabolites observed. During digestion triglycerides are primarily digested in the duodenum where pancreatic lipase hydrolyzes them into free fatty acids and monoglycerides, which are then combined with other lipid digestion products and bile salts to form micelles. These micelles are subsequently absorbed by the enterocytes. Similarly, the levels of glycerol detected in the final segment of the small intestines were low and there was no significant difference between groups as the glycerol may have been absorbed in the duodenum and jejunum due to their larger surface area, greater enzymatic activity, and longer exposure to bile salts and pancreatic lipases, which facilitate fat digestion. Furthermore, the low levels of cholesterol present in the meal suggest that most cholesterol detected during the study would have been linked to the endogenous trans-intestinal cholesterol efflux, the balance between *de novo* synthesis of cholesterol and its excretion from the gut into the intestines (24) for which Elevase® would have had little effect. Oats and wheat also have a relatively low protein composition [14 and 12 g for each 100 g (22)]. In these circumstances, it is likely that the native enzymes in the stomach and small intestinal tract were sufficient to adequately digest the protein present in the food matrix and Elevase® had little or no supplementary effect (Figure 4).

### 4.1 Limitations

The present study is not without limitations. Firstly, dietary food logs were not tracked but one assumes that within individuals there would not be profound differences of diet across the 4-week study. While participants were instructed not to change their exercise habits nor to engage in exercise 48 h prior to visiting the laboratory, we have no way of being completely sure participants followed these instructions or maintained a consistent level of physical activity for the duration of the study. Our sample size for the study was small as this demographic is difficult to recruit, yet there are some interesting findings in this small group that warrant further research. Future research should consider a longer supplementation duration to determine if we could detect stronger, physiologically relevant improvements in gastrointestinal readouts, and serum biomarkers associated with food digestion. Further, an investigation in clinical populations that experience food sensitivities may benefit from an accelerated metabolism of macronutrients that may be sensitizing the individuals. Alternatively, investigating if Elevase® accelerated breakdown of macronutrients in high-fat, high-protein or high-carbohydrate diets may also better inform the enzymatic capability of this dietary enzyme supplement.

### 4.2 Overall conclusions

To our knowledge this is the first placebo-controlled, crossover exploratory study using ileostomy samples to assess acute digestion of foods following dietary enzyme consumption (Figure 1). Overall, the findings in this study correlate well with an *in vitro* gut digestion model where Elevase® demonstrated accelerated breakdown of macronutrients in bread, pasta, and cereal within 2–3 h of starting the artificial digestion (8).

Consumption of Elevase® was well tolerated by participants (self-reported) and allowed for increased digestion of carbohydrates within 4 hours, in a small cohort of otherwise healthy individuals with a stable ileostomy. Given the known capabilities of enzymes to catabolize biomolecules in the diet, further placebo-controlled, randomized, double-blind studies with larger cohorts, longer duration of administration and different meal compositions are warranted.

## Data availability statement

The original contributions presented in the study are included in the article/supplementary material, further inquiries can be directed to the corresponding author.

## Ethics statement

The studies involving humans were approved by Clinical Research Ethics Committee of the Cork Teaching Hospitals. The studies were conducted in accordance with the local legislation and institutional requirements. The participants provided their written informed consent to participate in this study.

## Author contributions

SM: Data curation, Investigation, Writing – review & editing. AS: Data curation, Investigation, Writing – review & editing. JC: Project administration, Resources, Supervision, Writing – review & editing. EK: Writing – review & editing. MB: Supervision, Writing – review & editing. CP: Writing – review & editing. JD: Conceptualization, Writing – review & editing. KR: Conceptualization, Formal analysis, Supervision, Writing – original draft.
